# Ecotoxicity Assessment of α-Amino Acid-Derived Polyamidoamines Using Zebrafish as a Vertebrate Model

**DOI:** 10.3390/polym16142087

**Published:** 2024-07-22

**Authors:** Sofia Treccani, Paolo Ferruti, Jenny Alongi, Eugenio Monti, Daniela Zizioli, Elisabetta Ranucci

**Affiliations:** 1 Dipartimento di Chimica, Università degli Studi di Milano, Via C. Golgi 19, 20133 Milano, Italy; sofia.treccani@unimi.it (S.T.); paolo.ferruti@unimi.it (P.F.); jenny.alongi@unimi.it (J.A.); 2 Dipartimento di Medicina Molecolare e Traslazionale, Università degli Studi di Brescia, Viale Europa 11, 25123 Brescia, Italy; eugenio.monti@unibs.it

**Keywords:** ecocompatibility, fish embryo acute toxicity tests, flame-retardants, locomotor test, touch-evoked response test, water-soluble polymers, Zebrafish transgenic lines

## Abstract

The aquatic ecotoxicity of three α-amino acid-derived polyamidoamines (PAAs) was studied using zebrafish embryos as a viable vertebrate model organism. The PAAs examined were water-soluble amphoteric polyelectrolytes with a primarily negative charge, which were efficient flame retardants for cotton. The fish embryo acute toxicity test performed with PAA water solutions using 1.5–500 mg L^−1^ concentrations showed that toxicity did not statistically differ from the control. The survival rates were indeed >90%, even at the highest concentration; the hatching rates were >80%; and the numbers of morphological defects were comparable to those of the control. Tests using transgenic zebrafish lines indicated that the numbers of microscopic vascular and musculoskeletal defects were comparable to the control, with one random concentration showing doubled alterations. Sensory–motor tests in response to visual and tactile stimuli were also performed. In the presence of PAAs, embryos exposed to alternating light/dark cycles showed an insignificant mobility reduction during the dark phase. Touch-evoked response tests revealed a mild effect of PAAs on the neuromotor system at concentrations > 10 mg L^−1^. The cystine/glycine copolymer at 100 mg L^−1^ exhibited the greatest effect. Overall, the studied PAAs showed a minimal impact on aquatic systems and should be further considered as promising ecofriendly materials.

## 1. Introduction

Polyamidoamines (PAAs) are synthetic polymers prepared by the aza-Michael polyaddition of prim- or bis-sec-amines with bisacrylamides [1]. Almost all bisacrylamides and prim- or bis-sec-amines can be used as monomers, allowing the introduction of a wide variety of organic functions as pendants. Most of the PAAs studied so far are water-soluble, and the polymerization process preferably takes place at room temperature in water without added catalysts. Being a polyaddition reaction, the synthesis of PAAs does not involve the production of by-products. For all the above reasons, the synthetic process of PAAs can be considered green and easily scalable. Polyamidoamines are degradable in aqueous media [2], including physiological fluids, and are highly functional polymers that can be used for applications in different fields—for instance, as substrates for tissue engineering [3], carriers for gene therapy [4], and sorbents for water purification from heavy metal ions [5]. In addition, several bioinspired α-amino acid-derived PAAs have proved to be efficient flame-retardants (FRs) for cotton [6,7,8]. Given the widespread application of these polymers, this study investigates their ecotoxicity, aiming to provide a comprehensive assessment of their impact on aquatic ecosystems and contribute to safe polymer design. Water-soluble polymers rapidly impregnate the soil and dissolve quantitatively in surface and groundwater. Therefore, they represent a potential hazard for aquatic ecosystems since they may alter the physico-chemical properties of the aqueous matrix [9]. For instance, they can increase the bioavailability of persistent organic pollutants, facilitating their entry into the food chain [10]. Polyelectrolytes can particularly exhibit intrinsic toxicity due to their charge density [11] or promote the aggregation of organic substances [12] and minerals [13], forming floating dispersed matter. The harm of water-soluble polymers is often underestimated, although their REACH (regulation, evaluation, authorization, and restriction of chemicals; UE 1907/2006) registration is under discussion [11,14].

Zebrafish (*Danio rerio*) embryos represent a viable vertebrate animal model recommended by the European Experimental Substitution Research Center as a new alternative for in vivo testing in aquatic ecotoxicology [15]. Zebrafish share several biochemical pathways and anatomical and physiological features with mammals and humans [16,17,18]. Furthermore, they combine large-scale embryo production with rapid development, thus enabling a short-term assessment of latent effects and adverse outcomes in the response to early life exposure to environmental contaminants. Particularly in the flame-retardant technology sector, zebrafish embryos have been used in the toxicity profiling of numerous flame-retardants used in various consumer products, including plastics, textiles, protective clothing, furniture, automotive and electronic products [19,20,21].

Based on this premise, this study investigates the aquatic ecotoxicity of three representative PAAs derived from α-amino acids using zebrafish embryos as a vertebrate model [22]. All three polymers are amphoteric, predominantly anionic, water-soluble, and act as efficient flame retardants for cotton. Their ecotoxicological assessment was based on different types of toxicological tests. The fish embryo acute toxicity test and the analysis of microscopic phenotypic abnormalities induced by exposure of genetically modified embryos to PAAs provided a general overview of the short-term toxicity of these polymers. Neurotoxicological tests based on the evaluation of the response to light and tactile stimuli allowed us to study the effect of different PAA doses on the locomotor development of embryos. This study is believed to be the first of its kind to explore the in vivo aquatic ecocompatibility of this family of polymers. It could, therefore, represent a significant milestone in the design of new environmentally friendly polymeric flame retardants.

## 2. Materials and Methods

### 2.1. Materials

Glycine (coded as GLY, 98%), glutamic acid monohydrate (GLU, >98%), cystine (CYSS, >98.0%), N,N′-methylenebisacrylamide (MBA, 99%), lithium hydroxide monohydrate (98%), and 6 M HCl were supplied by Sigma-Aldrich (Milan, Italy) and used as received.

### 2.2. Synthesis of PAAs

The PAA-encoded M-GLY was synthesized as already reported [23]. In brief, MBA (2.01 g; 0.013 mol), glycine (0.97 g; 0.013 mol), and lithium hydroxide monohydrate (0.55 g; 0.013 mol) were suspended in water (5.0 mL). The reaction mixture was warmed up to 45 °C under stirring until complete dissolution and then left for 4 days at 25 °C in the dark in inert nitrogen atmosphere. The pH of the reaction medium was around 10.5. The resultant viscous homogeneous solution was diluted to 50 mL with water, acidified to pH 4.5 with a few drops of 6 M HCl, and finally ultrafiltered through an Amicon^®^ Millipore (Millipore, Milan, Italy) ultrafiltration system using a regenerated cellulose membrane with nominal molecular weight cut-off of 3000. The final product was retrieved by freeze-drying.

The PAA-encoded M-GLU was synthesized and purified by following the same procedure described for M-GLY using MBA (2.00 g, 0.013 mol), L-glutamic acid (1.91 g, 0.013 mol), lithium hydroxide monohydrate (1.11 g, 0.026 mol), and water (6.0 mL). The reaction time was 9 days. The pH of the reaction medium and of the ultrafiltered M-GLU solution were 10.5 and 4.5, respectively.

The PAA-encoded M-GLY_50_-CYSS_50_ was synthesized by following the same procedure described for M-GLY, using MBA, glycine, L-cystine, and lithium hydroxide monohydrate in a 1:0.5:0.5:1.5 molar ratio: MBA (2.19 g, 14.2 mmol), glycine (0.53 g, 7.1 mmol), L-cystine (1.71 g, 7.1 mmol), lithium hydroxide monohydrate (0.90 g, 21.4 mmol), and water (8.0 mL). The reaction time was 2 days. The pH of the reaction medium and of the ultrafiltered M-GLY_50_-CYSS_50_ solution were 10.5 and 8.5, respectively. Below the latter pH, the water solubility of M-GLY_50_-CYSS_50_ was drastically reduced.

### 2.3. Biological Experiments

#### 2.3.1. Ethics Statement

Experiments took place at the Zebrafish Facility, Department of Molecular and Translational Medicine, University of Brescia, Italy. All animal experiments were conducted in accordance with the Italian and European regulations on animal care and the standard rules defined by the Local Committee for Animal Health (OPBA) and authorized by the Italian Ministry of Health (Authorization Number 585/2018).

#### 2.3.2. Zebrafish Maintenance and Egg Collection

Zebrafish (*Danio rerio*) embryos were collected from the AB wild-type line and two transgenic lines: Tg(*fli1*:EGFP) and Tg(*Bmp*:EGFP).

In Tg(*fli1*:EGFP) strain, the enhanced green fluorescent protein (EGFP) is placed upstream of the *fli1* gene and expressed in endothelial cells, which are related to blood vessel growth and vasculature formation during embryogenesis [24,25]. In this work, the fluorescent line Tg(*fli1*:EGFP) was used to monitor in vivo embryonic angiogenesis and evaluate the potential vasotoxicity and cardiotoxicity of the investigated PAAs.

In Tg(*Bmp*:EGFP) line, the enhanced green fluorescent protein (EGFP) is expressed under the BMP Response Element [26,27]. BMPs are a family of signaling proteins involved in several key processes, such as tissue differentiation, development of tail bud, cardiovascular system, and somite formation. The fluorescent line Tg(*Bmp*:EGFP) was used to monitor in vivo the embryonic musculoskeletal system and evaluate the effect of the investigated PAAs on its early developmental stage.

Adult fishes were maintained under standard laboratory conditions, as previously described [28], in a recirculating aquaculture system (Techniplast ZebTEC, Buguggiate, Varese, Italy) at 27 ± 1 °C in a 14 h light and 10 h dark daily cycle. The housing system guarantees fish water (0.1 g L^−1^ of Instant Ocean Sea Salts, 0.1 g L^−1^ of sodium bicarbonate, and 0.19 g L^−1^ of calcium sulfate) at constant pH and conductivity values; ammonia, nitrite, and nitrate were kept below detection limits (0–5, 0.025–1, and 0–140 mg L^−1^, respectively). Adult male and female animals were mated in the breeding box overnight. The next morning, freshly spawned eggs were collected, washed with fresh fish water, and maintained at 28 °C in 10 cm Ø Petri dishes containing fresh fish water until the onset of gastrulation, i.e., 4 h post-fertilization (hpf), according to the literature [29], and finally exposed to the substance. Egg batches were used only when fertilization rates were ≥80%.

#### 2.3.3. Fish Embryo Acute Toxicity (FET) Test

Fish embryo acute toxicity test (FET) was carried out according to the Organization for Economic Co-operation and Development (OECD) guideline 236 [30]. The FET test is intended to determine the acute or lethal toxicity of chemicals and is normally carried out using AB wild-type embryos.

Stock PAA solutions with concentration of 1 g L^−1^ were first prepared in fish water. Toxicity tests were carried out in freshly prepared PAA solutions obtained by diluting the stock solutions with fish water at eight concentrations, namely, 1.5, 2, 5, 10, 25, 50, 100, and 500 mg L^−1^. Tests were conducted by progressively doubling the concentration of water PAA solutions to which zebrafish were exposed. Since the effects of PAAs were not particularly evident up to 100 mg L^−1^, it was decided to significantly increase the concentration up to 500 mg L^−1^ to intensify the stress to which zebrafish were subjected.

Tests were performed in triplicate using 25–30 alive embryos per tested concentration. Embryo exposure was performed in a Petri dish using the so-called static immersion method from 4 hpf (gastrula stage) [31]. Fish water was used as a negative control (embryonic mortality ≤10%) and a 3,4-dichloroaniline solution (3.7 mg L^−1^) as a positive control (embryonic mortality ≥90%).

Since, until 48 hpf, embryos are enclosed in the chorion membrane, a porous acellular membrane with limited permeability to high-molecular-weight organic species [32,33,34,35], to avoid the chorion’s barrier function and allow for maximum uptake of the analyzed substances, the OECD guideline 236 [30] recommends an exposure duration ≥ 96 hpf [36]. In this work, the exposure duration adopted was indeed 120 hpf. Always according to the OECD guideline 236, embryos were observed regularly (48, 72 and 120 hpf), and their survival rate, calculated as in Equation (1), was monitored by adopting the coagulation of fertilized eggs and lack of heartbeat as indicators of lethality. Dead embryos were removed at each observation time.

(1) $$Survival\;rate=\frac{survived\;fish}{total\;fish}\times 100$$

Spontaneous dechorionation (hatching) of fertilized eggs normally occurs from 48 hpf (larva stage) to 72 hpf. The hatching rate, calculated as in Equation (2), was determined at 72 hpf.

(2) $$Hatching\;rate=\frac{hatched\;embryos}{total\;exposed\;eggs}\times 100$$

Additionally, at 72 hpf, hatched embryos exposed to the different PAA doses were anesthetized with 0.4% tricaine (Merck group, Milan, Italy), placed on their sides in 1% agarose, and their morphologies were inspected using a Zeiss Axiozoom V13 microscope (Carl Zeiss AG, Oberkochen, Germany) equipped with a PlanNeoFluar Z 1X/0.25 FWD 56 mm lens. After observation, embryos were immersed again in the original PAA solution (or fish water). The percentage of malformed larvae (spinal cord malformation, pericardial edema, and growth retardation) was compared to that observed in the negative control.

#### 2.3.4. Transgenic Tg(*fli1*:EGFP) Zebrafish Embryos for Angiogenesis Assessment

Angiogenesis was monitored in transgenic Tg(*fli1*:EGFP) embryos at 30 hpf by visualizing the development of intersegmental vessels (ISVs), the first hallmark of angiogenesis, which appeared as green fluorescent. Tests were performed in triplicate using 25–30 alive embryos per tested concentration. Embryos were exposed to 2 and 10 mg L^−1^ PAA solutions at 4 hpf. They were subsequently dechorionated manually at 30 hpf prior to examination using watchmakers’ forceps (Damon n° 5) according to a reported procedure [37]. They were then anesthetized with 0.4% tricaine and observed using a Zeiss Axiozoom V13 fluorescence microscope (Carl Zeiss AG, Oberkochen, Germany) equipped with a PlanNeoFluar Z 1X/0.25 FWD 56 mm lens. Fluorescent images depicting the vascular tree of both control and treated groups were acquired in a lateral position at 63X magnification and processed using the Zen 3.5 (Carl Zeiss AG, Oberkochen, Germany) software.

#### 2.3.5. Transgenic Tg(*Bmp*:EGFP) Zebrafish Embryos for Somite Segmentation Assessment

Somite segmentation was monitored in transgenic Tg(*Bmp*:EGFP) at 48 hpf by visualizing green fluorescent somites, the first hallmark of the musculoskeletal system.

Tests were performed in triplicate using 25–30 alive embryos per tested concentration. Embryos were exposed to 2 and 10 mg L^−1^ PAA solutions at 4 hpf. At 48 hpf, prior to examination, they were dechorionated manually, as described for Tg(*fli1*:EGFP). They were then anesthetized with 0.4% tricaine and observed using the same apparatus described for Tg(*fli1*:EGFP). Fluorescent images depicting somite development of both control and treated groups were acquired in a lateral position at 40X magnification and processed using the Zen 3.5 software (Carl Zeiss AG, Oberkochen, Germany).

#### 2.3.6. Locomotor Behavior Assessment with the Light/Dark Locomotion Test

The locomotor behavior was assessed by the light–dark locomotion test [38]. AB-strain embryos at 4 hpf were exposed to different PAA concentrations (1.5, 2, 5, 10, 25, 50, and 100 mg L^−1^) and fish water as control until 120 hpf using the immersion method. Larvae were then transferred into a 96-square well plate, placing a single larva per well containing 200 μL of fish water. The locomotion experiments were carried out following the guidelines included in “Zebrafish protocols for neurobehavioral research” [39], which recommend that experiments be carried out on 36 larvae for each PAA concentration. The plate was then placed in the observation chamber of a DanioVision (Noldus, Wageningen, The Netherlands) system holder in an isolated and noise-free room and at 28 °C. The system was set up to track by video recording movements of each single larva under light/dark stimuli. The experimental protocol consisted of 30 min light acclimatization followed by 3 alternating dark/light cycles lasting 10 min per type of exposure (Figure S1 in Supplementary Materials). Movements were analyzed using the Noldus Ethovision software (Version XT 13.0, Noldus, Wageningen, The Netherlands) and reported as total traveled distance (mm).

#### 2.3.7. Touch-Evoked Response Test

AB-strain embryos were exposed at 4 hpf to different PAA concentrations (1.5, 2, 5, 10, 25, 50, and 100 mg L^−1^) and fish water as control until 72 hpf using the immersion method. For each treatment, tests were performed in triplicate on 25 larvae, for a total of 75 larvae. Each single larva was then placed in the center of a Petri dish containing the test solution. Below the dish, a motility wheel consisting of two concentric circles of increasing diameter, namely, 10 mm and 20 mm, was placed, and a microscope was centered. The tail of the larva was gently touched with a smooth pipette tip, and the touch response was observed and categorized as follows: (1) larvae that did not move, i.e., that remained within the circle with 10 mm diameter; (2) larvae that swam between the circles with 10 and 20 mm diameters; and (3) larvae that swam across the outer circle with 20 mm diameter.

#### 2.3.8. Statistical Analysis

All the experiments were performed in triplicate for both the control and the treated groups. Data were expressed as mean ± standard deviation. OriginPro 2019 software (Adalta, Arezzo, Italy) was used for the statistical analysis of the survival and hatching rate, as well as the light–dark locomotion test. The data distribution was evaluated for normality by the Shapiro–Wilk test. Data from survival and hatching rates were analyzed with one-way analysis of variance (ANOVA) followed by Bonferroni post hoc test. Pairwise comparisons between each treated group and the control group of light–dark locomotion test were performed by the unequal variances Welch’s *t*-test. All tests were two-tailed, and a significance level *α* = 0.05 was considered; that is, all data with *p* < 0.05 were considered statistically significant.

## 3. Results and Discussion

### 3.1. Polyamidoamines (PAAs): Synthesis and Ionic Species Distribution

**Figure 1:**
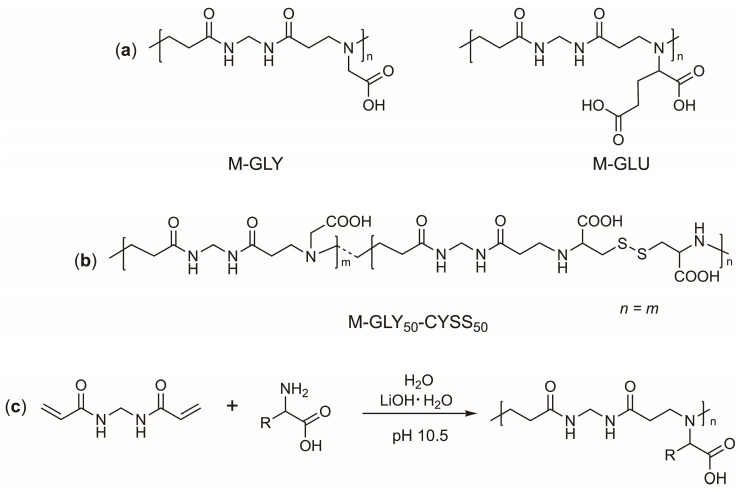
Structures of the repeat units of (**a**) M-GLY and M-GLU and (**b**) M-GLY_50_-CYSS_50_; (**c**) synthesis of α-amino acid-derived PAAs.

The aim of this work was to study the ecocompatiblity of three α-amino acid-derived polyamidoamines (PAAs) that have recently proved to be efficient flame retardants (FRs) for cotton. These PAAs, encoded M-GLY, M-GLU, and M-GLY_50_-CYSS_50_ (Figure 1a,b), contain carboxyl- and tert-amine groups in their repeat units. Therefore, they are amphoteric polyelectrolytes featuring peculiar ionic species distributions.

All PAAs were synthesized according to a classical procedure by the aza-Michael polyaddition of N,N′-methylenebisacrylamide (MBA) with glycine, glutamic acid, and a 1:1 molar ratio glycine/cystine mixture, respectively (Figure 1c) [23,40]. All samples were obtained in a one-pot process carried out in a water solution at a pH of 10.5 and at a total solid concentration from 40 to 50 wt.%.

Due to the different steric hindrance of the side substituents, the primary amine groups of the α-amino acids have different reactivities in the 1,4-addition to the acrylamide functions of MBA and require different reaction times (see Section 2.2). PAAs were purified by ultrafiltration through membranes with a molecular weight cut-off of 3000 to eliminate the oligomeric fractions and finally retrieved by freeze-drying. Their chemical structures were confirmed by ^1^H-NMR spectroscopy (Figures S2–S4, respectively, in Supplementary Materials). The average molecular weights were calculated from the ^1^H-NMR spectra from the ratio between the integrals of the resonance peaks relative to the internal units and the integrals of the resonance peaks relative to the terminal repeat units. The spectra were consistent with molecular weights higher than 10,000 for all PAAs.

The ionic species distributions of M-GLY, M-GLU, and M-GLY_50_-CYSS_50_ shown in Table 1 were determined from the speciation curves shown in Figures S5–S7 obtained from the *pK_a_* values of the ionizable functions present in them. The calculated isoelectric points (*IP*) are consistent with the presence in their repeat units at a pH of 7.0 (a pH of 7.5 in the case of M-GLY_50_-CYSS_50_) of an overall negative charge. M-GLY and M-GLY_50_-CYSS_50_ are moderately anionic, whereas M-GLU is highly anionic. The first two feature 39% and 35% repeat units bearing a single net negative charge, respectively, the remaining being zwitterionic. The net average charge per repeat unit is −0.39 and −0.35, and the positive/negative charge ratio is 0.61 and 0.72, respectively. M-GLU features 14% repeat units with two negative charges, the remaining bearing two negative charges and one positive charge. The net average charge per repeat unit is −1.14, and the positive/negative charge ratio is 0.43.

### 3.2. Timeline of the Experiments Carried Out on Zebrafish Embryos Exposed to PAAs

**Figure 2:**
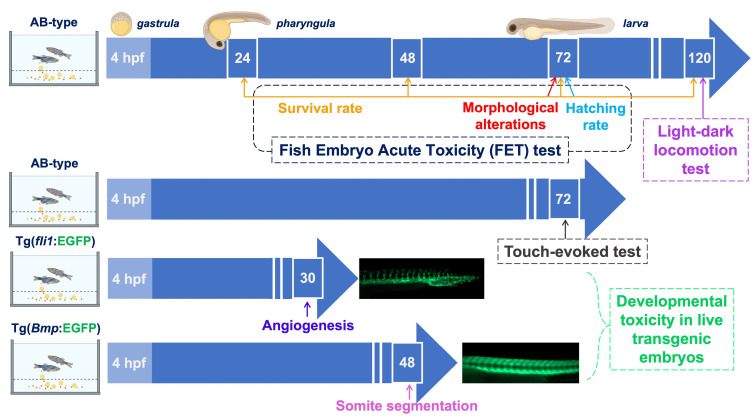
Timeline of the ecotoxicity tests carried out on PAA-exposed zebrafish embryos. Figures in the white frames represent the execution times; hpf stands for hours post-fertilization. From the top: 1st row: tests performed on AB wild-type embryos. In the FET test, developmental toxicity was assessed using different endpoints: survival rate, morphological alterations, and hatching rate. In the light/dark locomotion test, the response of zebrafish embryos’ locomotion activity to a sudden transition from light to darkness was assessed. 2nd row: touch-evoked response test performed on AB wild-type larvae. 3rd row: tests performed on Tg(*fli1*:EGFP) transgenic embryos expressing enhanced green fluorescent protein. The angiogenetic process was monitored by observing the development of intersegmental vessels. 4th row: tests performed on Tg(*Bmp*:EGFP) transgenic embryos expressing enhanced green fluorescent protein. The somite-segmentation process was monitored by observing the correct development of somites in a V-shaped pattern.

The temporal sequence of the tests performed on zebrafish embryos and the endpoints considered is shown in Figure 2.

### 3.3. Fish Embryo Acute Toxicity (FET) Test

**Figure 3:**
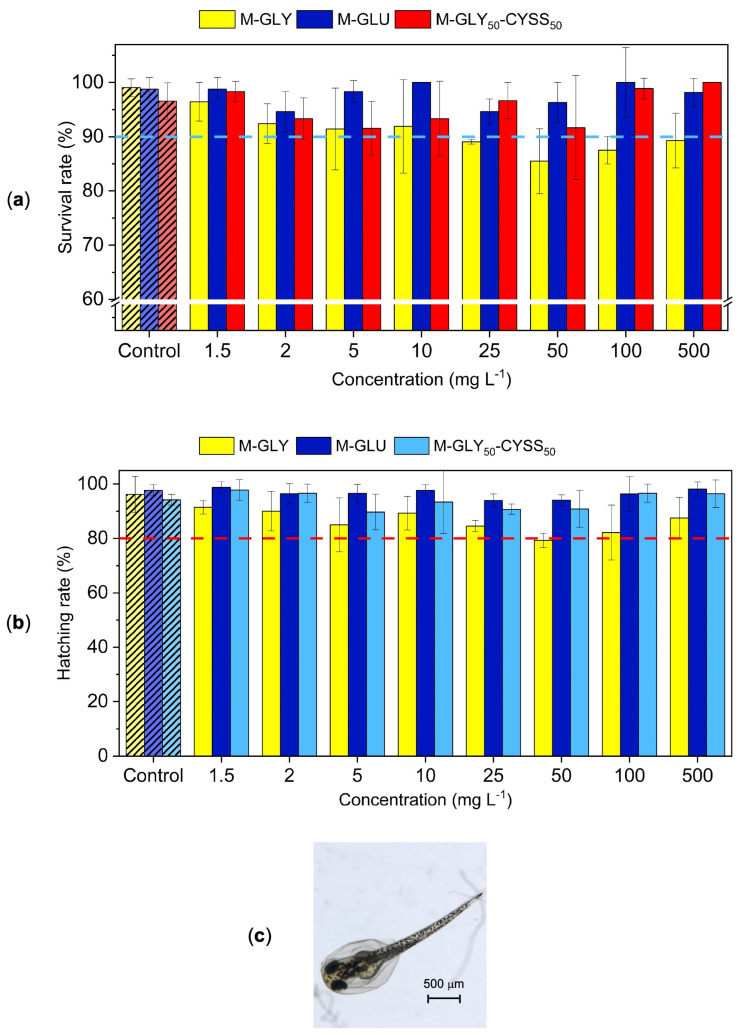
(**a**) Survival rate of AB wild-type larvae after exposure to different PAA concentrations for 120 hpf. The dotted blue line represents the threshold value for the negative control indicated by the OECD guideline 236 [30] to assess the test validity; (**b**) hatching rate of AB wild-type larvae after exposure to different PAA concentrations for 72 hpf; (**c**) bright-field microscopic image of a hatching zebrafish larva at 72 hpf. In (**a**,**b**), controls represent larvae exposed to fish water. For each PAA dose, data were obtained from three independent experiments and expressed as mean ± standard deviation. The dotted red line represents the threshold value for the negative control indicated by the OECD guideline 236 to assess the test validity. The absence of asterisks indicates that no statistically significant differences were observed compared to control (significance level *α* = 0.05).

**Figure 4:**
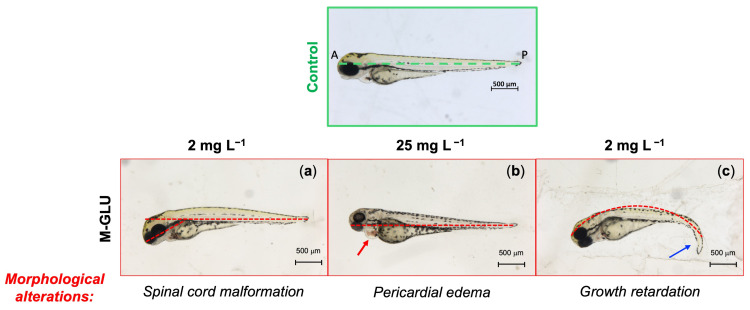
Representative bright-field microscopic images of morphological alterations by FET test observed in zebrafish larvae after 72 hpf exposure to M-GLU solutions. The dashed green line in the control represents the anterior–posterior A-P axis. (**a**) Spinal cord malformation (2 mg L^−1^), revealed by the abnormal angle of the head relative to the A-P axis; (**b**) pericardial edema (25 mg L^−1^), indicated by the red arrow; (**c**) growth retardation and defects in anterior–posterior axis (2 mg L^−1^), revealed by the abnormal trunk alignment (dashed red line) and tail deformation (blue arrow). All pictures are lateral views with dorsal at the top (magnification 32X).

**Figure 5:**
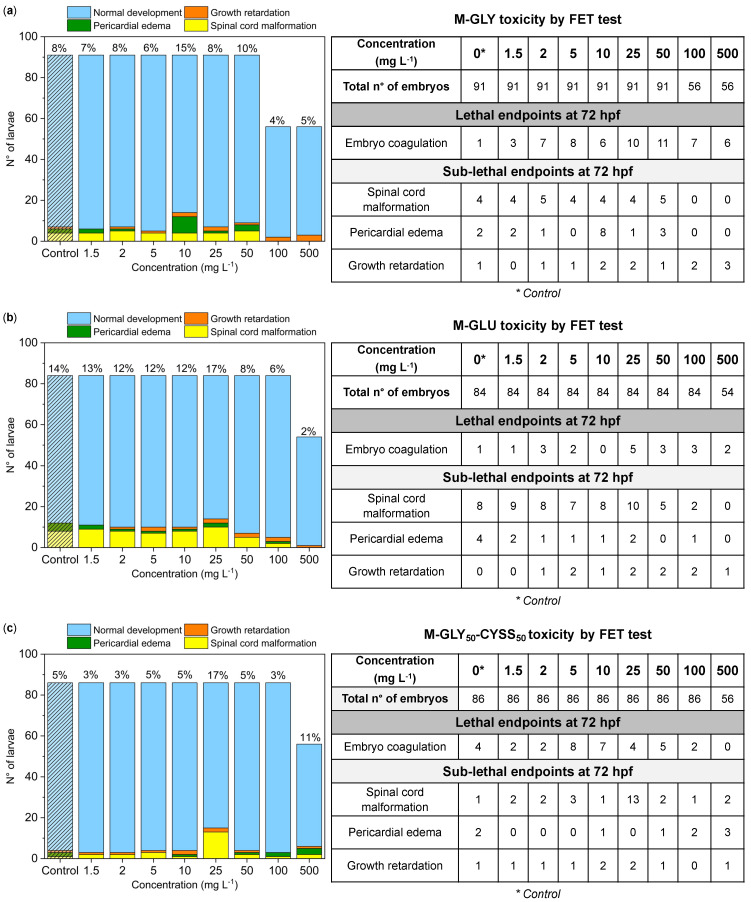
Morphological alterations of zebrafish larvae observed in the FET test at 72 hpf after exposure to (**a**) M-GLY, (**b**) M-GLU, and (**c**) M-GLY_50_-CYSS_50_. For each PAA concentration, the total number of embryos is the sum of embryos tested in three independent experiments. Sub-lethal endpoints are color-coded: green bars represent pericardial edema, red bars growth retardation, and yellow bars spinal distortion. Control represents zebrafish embryos exposed to fish water.

The acute toxicity of M-GLY, M-GLU, and M-GLY_50_-CYSS_50_ was assessed using the FET test on AB wild-type embryos, following the OECD test guideline 236 [30]. Several lethal and sub-lethal endpoints [43] were monitored at well-defined time points, namely, the survival rate, macroscopic morphological alterations, and the hatching rate (Figure 2). The collected data are shown in Figure 3, Figure 4 and Figure 5 and in Figures S8–S11.

Embryos were observed at 48, 72, and 120 hpf with the aim of detecting the time dependence of zebrafish embryo viability. A lack of heartbeat and coagulation, identified by the milky appearance of embryos when visually observed and by the dark color when viewed under bright-field optical microscopy, were indicators of lethality. The survival rate of zebrafish embryos exposed to different PAA concentrations (1.5, 2, 5, 10, 25, 50, 100, and 500 mg L^−1^) was expressed as the percentage of live embryos over the total number of tested embryos. For each PAA concentration and time, the mortality was compared with that of the negative control (fish water). The results obtained at 120 hpf are shown in Figure 3a, whereas Figure S8a,b features the trend observed at 48 to 72 hpf. The embryo survival rate in the negative control was ≥90% until the end of the exposure, in line with the OECD test guideline 236 [30]. Noteworthy, the survival rate of all PAAs did not statistically differ from that of the negative control. The ecotoxicity levels of chemicals can be classified based on the value of the lethal dose 50, LC_50_, defined as the concentration required to kill 50% of embryos as dangerous (10 mg L^−1^ < LC_50_ < 100 mg L^−1^), toxic (1 mg L^−1^ < LC_50_ < 10 mg L^−1^), and carcinogenic (LC_50_ < 1 mg L^−1^) substances [44]. All tested PAAs could be therefore classified as non-dangerous, non-toxic, and non-carcinogenic since their survival rate at 120 h was >>50%, even at 500 mg L^−1^, i.e., up to a level which could only be found in the environment in the event of a massive spill.

The OECD test guideline 236 [30] recommends monitoring the hatching rate of zebrafish embryos for all treatments and controls from 48 to 72 hpf as a key parameter to ensure embryo exposure to chemicals while avoiding the chorion barrier effect. It is generally recognized that low hatchability can be associated with the delayed growth of embryos and is classified as a relevant sublethal endpoint [44]. However, zebrafish embryos can also prematurely hatch under the effect of chemicals [45], resulting in the underdeveloped swimming ability of larvae. Overall, neither early nor delayed hatching was observed under the effect of PAAs. The hatching rate values obtained at 72 hpf (larva stage) using Equation (2) (Section 2.3.3) are shown in Figure 3b, where the effect of PAAs on hatching is compared with that of the negative control. According to the OECD test guideline 236, the hatching rate in the negative control should be ≥80% at the end of 96 hpf exposure. In this work, the hatching rate of the control was ≥90% already at 72 hpf, and, most relevant, the hatching rate was not affected by PAA treatments over the entire concentration range for all PAAs.

Other relevant sub-lethal endpoints considered include morphological alterations, such as spinal cord malformation, pericardial edema, growth retardation, as well as missing pigmentation, and yolk deformation, all monitored at 72 hpf. Among them, missing pigmentation and yolk deformation were never detected. Examples of morphological alterations detected after exposure to M-GLU for 72 hpf are shown in Figure 4. Their incidence in relation to embryo exposure to PAAs is reported in Figure 5a–c, where the number of larvae with morphological alterations is plotted versus the test concentration. Morphological alterations of zebrafish larvae exposed to all PAA concentrations are shown in Figures S9–S11.

In the case of M-GLY exposure (Figure 5a), a significant number of morphological alterations with respect to the control was observed only at the intermediate concentration of 10 mg L^−1^, which presented the greatest number of pericardial edema malformations (8 vs. 2 in the control). Overall, no dose dependence could be identified in the embryo response to M-GLY exposure. In the case of both M-GLU and M-GLY_50_-CYSS_50_ exposure (Figure 5b,c), an increase in the total number of malformations was observed only at 25 mg L^−1^ (17% vs. 14% in the control for M-GLU, and 17% vs. 5% in the control for M-GLY_50_-CYSS_50_). At this concentration, the greatest number of spinal cord malformations was indeed presented in both cases. In conclusion, it may be observed that the sub-lethal endpoints at 72 hpf were not relevant and almost independent of the PAA doses. The few random concentrations inducing a significantly different response compared to the negative control were reasonably due to the variability of the animal model.

### 3.4. Analysis of Microscopic Malformations Using Transgenic Zebrafish Lines

#### 3.4.1. Fluorescent Tg(*fli1*:EGFP) Zebrafish Lines for the Assessment of the Angiogenesis Process during Embryonic Development

**Figure 6:**
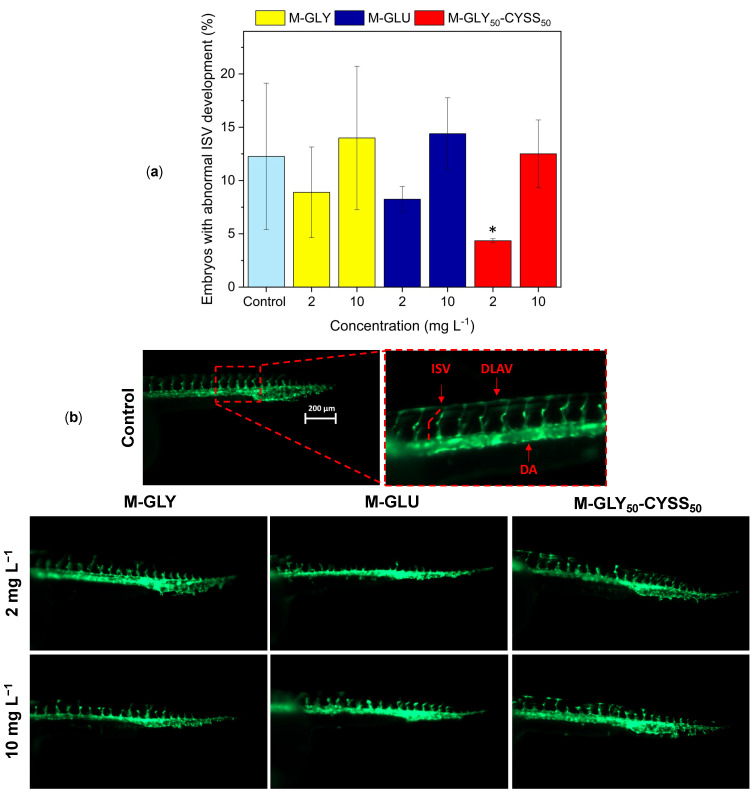
Intersegmental vessel (ISV) alteration in PAA-treated Tg(*fli1*:EGFP) embryos: (**a**) average number of zebrafish embryos exposed for 30 hpf to 2 and 10 mg L^−1^ of M-GLY (yellow), M-GLU (blue), and M-GLY_50_-CYSS_50_ (red) with ISV alterations. For each PAA dose, data were obtained from three independent experiments and expressed as mean ± standard deviation. The asterisk indicates significant differences (*p* < 0.05) compared to control. (**b**) Representative lateral views of control and PAA-treated embryos with ISV alterations (magnification 63X). ISVs are labeled with a green fluorescent marker. Brightness of images was increased by 40%. DLAV = dorsal longitudinal anastomotic vessel; DA = dorsal aorta.

The small size and transparency of zebrafish embryos allow real-time and in vivo observation of intracellular organelles, tissues, or even cells using transgenic fluorescent lines [46,47,48]. In Tg(*fli1*:EGFP) embryos, in particular, the enhanced green fluorescent protein (EGFP) is placed upstream of the *fli1* gene and is expressed in endothelial cells, which are related to vasculature formation during embryogenesis. The first hallmark of angiogenesis is the formation of new intersegmental vessels (ISV) (Figure 6b) that sprout from the aorta (16 hpf), run between each pair of somites (26–30 hpf), and connect to the dorsal longitudinal anastomotic vessel (DLAV) becoming part of the trunk vasculature (48 hpf).

In this study, Tg(*fli1*:EGFP) embryos were treated at 4 hpf with two intermediated PAA doses, namely, 2 and 10 mg L^−1^, then dechorionated at 30 hpf and finally optically inspected. It should be observed that the hydrodynamic radii of α-amino acid-derived PAAs are in the order of 1–4 nm [49,50,51]. Therefore, since pores distributed on the chorionic membrane have diameters from 0.5 to 0.7 μm at 4 hpf [35,52], it is reasonable to assume that PAA solutions permeate the chorion. Figure 6b illustrates the ISV alterations observed at 30 hpf due to PAA exposure, while Figure 6a displays the average number of embryos exhibiting these alterations at the tested concentrations. The data demonstrate that PAA treatment did not significantly alter the early development of angiogenesis in zebrafish embryos since the number of the observed ISV alterations did not statistically differ from those presented by the negative control, irrespective of the PAA structure and dose.

#### 3.4.2. Fluorescent Tg(*Bmp*:EGFP) Zebrafish Lines for the Assessment of Somite Segmentation

Transgenic Tg(*Bmp*:EGFP) zebrafish embryos were used to study the expression, localization, and regulation of bone morphogenetic protein (BMP) genes by using the EGFP green fluorescent marker. BMPs are a family of signaling proteins involved in several key processes, such as tissue differentiation, development of tail bud, brain structures, cardiovascular system, and somite formation, which give rise to the axial skeleton and the skeletal muscle of the trunk [53,54]. In zebrafish embryos, somites develop into a bilaterally symmetrical V-shaped sequence in an anterior-to-posterior direction. Physical boundaries separate each somite from the adjacent one [55]. Trunk malformation should be one of the most relevant deformities normally observed in zebrafish embryos, which affects their locomotor capacity. Segmentation begins at approximately 10.5 hpf and completes at 24 hpf.

**Figure 7:**
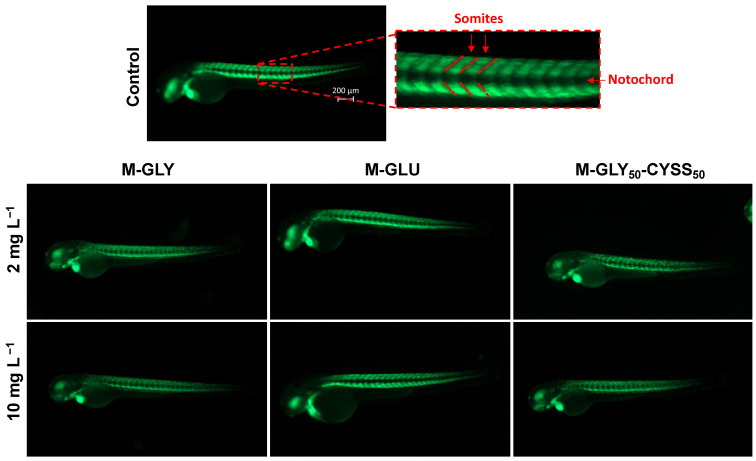
Representative lateral views of control and PAA-treated Tg(*Bmp*:EGFP) embryos (magnification 40X). Somites are labeled with a green fluorescent marker. Brightness of images was increased by 40%.

In this experiment, somite development in embryos exposed to 2 and 10 mg L^−1^ PAA solutions was assessed at 48 hpf, i.e., when BMP expression reached its maximum level. Embryos exposed to fish water represented the negative control (Figure 7). In all experiments, the anterior to posterior wave reached a total of about 30 V-shaped somites, made of perfectly symmetric pairs. No altered axial curvature was observed, as shown by the well-aligned notochord line; the rod-like structure formed on the dorsal side. It should be noted that, in another study, clear anomalies in body flexion were observed after 24 hpf in the presence of highly toxic substances, such as 100 mM cadmium ions, which included loops, bends, and swelling of the notochord [56].

Analogously to what was observed in experiments with Tg(*fli1*:EGFP) zebrafish lines, PAA treatment did not significantly alter the somite segmentation of zebrafish embryos.

### 3.5. Sensory–Motor Tests

The sensory–motor tests assess the sensory perception of zebrafish, including visual, auditory, or tactile stimuli, and the subsequent and spontaneous motor response [57]. In this study, AB wild-type embryos underwent two types of sensory–motor tests, one based on light stimuli and the other on tactile stimuli.

#### 3.5.1. Locomotor Behavior Assessment Using the Light/Dark Locomotion Test

Zebrafish embryos have been extensively studied to correlate behavioral patterns with the effects of exposure to chemicals on brain development [38,58]. In particular, light and dark stimuli-induced locomotor behavior is a widespread behavioral model used to study neuroactive compounds [59]. Zebrafish larvae show mature swimming at 120 hpf, following the development of the swim bladder [60], and exhibit a specific pattern of movement when exposed to alternating light and dark conditions [61]. The light/dark transition increases their locomotor activity, while the dark/light transition decreases it, in conjunction with increased stress/anxiety levels in larvae.

**Figure 8:**
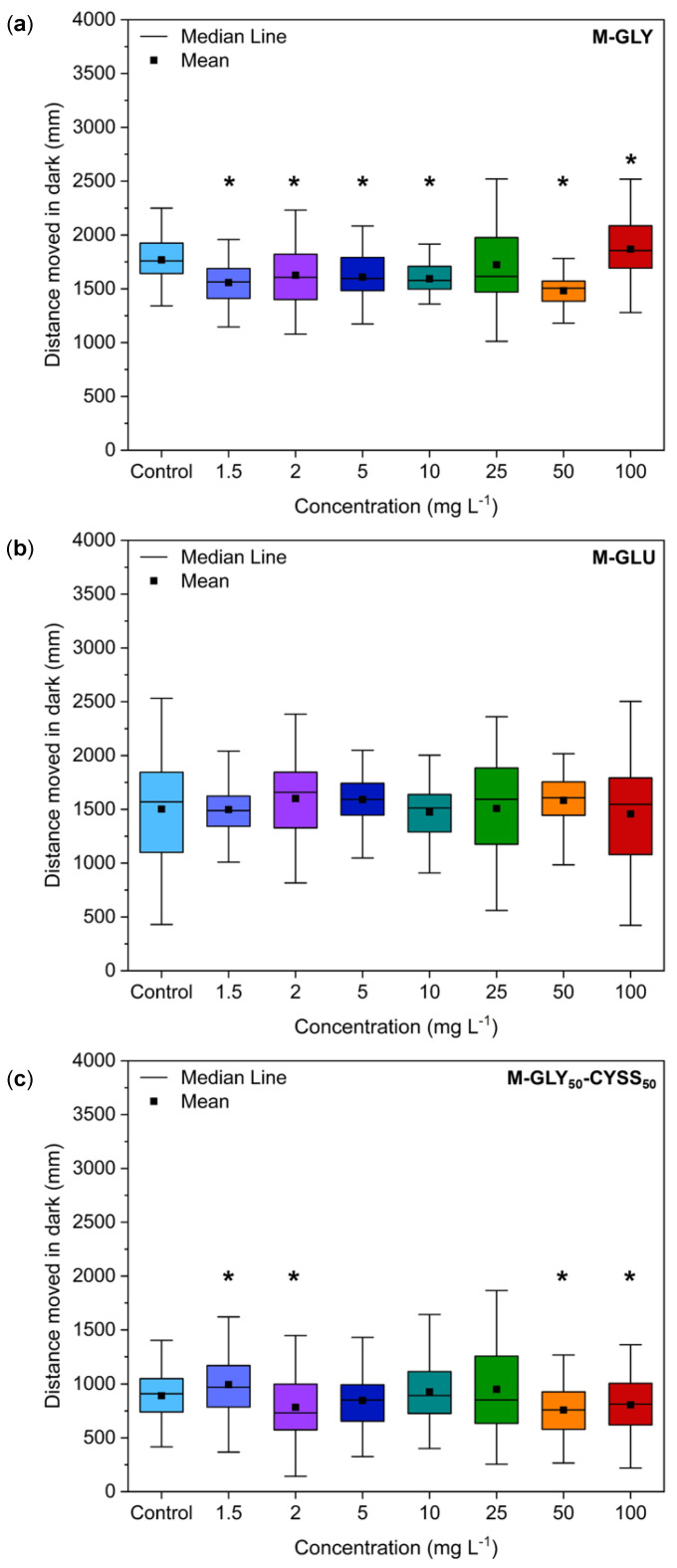
Results of the locomotion tests performed using AB-wild-type embryos at 120 hpf. Distance moved by zebrafish larvae exposed to different M-GLY (**a**), M-GLU (**b**), and M-GLY_50_-CYSS_50_ (**c**) concentrations during dark periods. Data were collected with n = 36 larvae per concentration. Data distribution is represented by the box-and-whisker plot. Asterisks indicate significant differences (*p* < 0.05) compared to control.

In this study, the locomotor activity of zebrafish larvae exposed to PAAs was analyzed at 120 hpf. The average total distance swum during dark periods is shown in Figure 8a, Figure 8b, and Figure 8c for M-GLY, M-GLU, and M-GLY_50_-CYSS_50_, respectively. It may be observed that a slight significantly different decrease in the overall distance traveled compared to the negative control was induced by M-GLY exposure over the entire concentration range, apart from at 25 mg L^−1^. A slightly significant different decrease in the average distance traveled compared to the negative control was induced by M-GLY_50_-CYSS_50_ for treatments at 1.5, 2 mg L^−1^, 50 mg L^−1^, and 100 mg L^−1^. In contrast, M-GLU exposure did not affect the locomotor behavior of zebrafish larvae over the entire concentration range up to 100 mg L^−1^. No significant dose dependence was observed over the entire concentration range. Overall, the results collected confirmed the substantial mild aquatic toxicity of PAAs resulting from the FET tests and experiments on transgenic lines.

#### 3.5.2. Touch-Evoked Response Test

In the touch-evoked response test, the response of zebrafish embryos to a sudden tactile stimulus, such as an unexpected and sudden touch on the tail, was monitored [57]. The tactile startle response is present in zebrafish embryos as early as at 2 dpf [62] and is extremely fast. It starts within approximately 5 ms of the stimulus onset [57]. During the startle, the fish bends so that the head touches the tail (C-start response) in approximately 15 ms and then moves several body lengths away from its starting location in 100 ms. It should be observed that the correct development of the spinal cord plays a critical role in touch-evoked responses by integrating sensory inputs and generating appropriate motor response.

**Figure 9:**
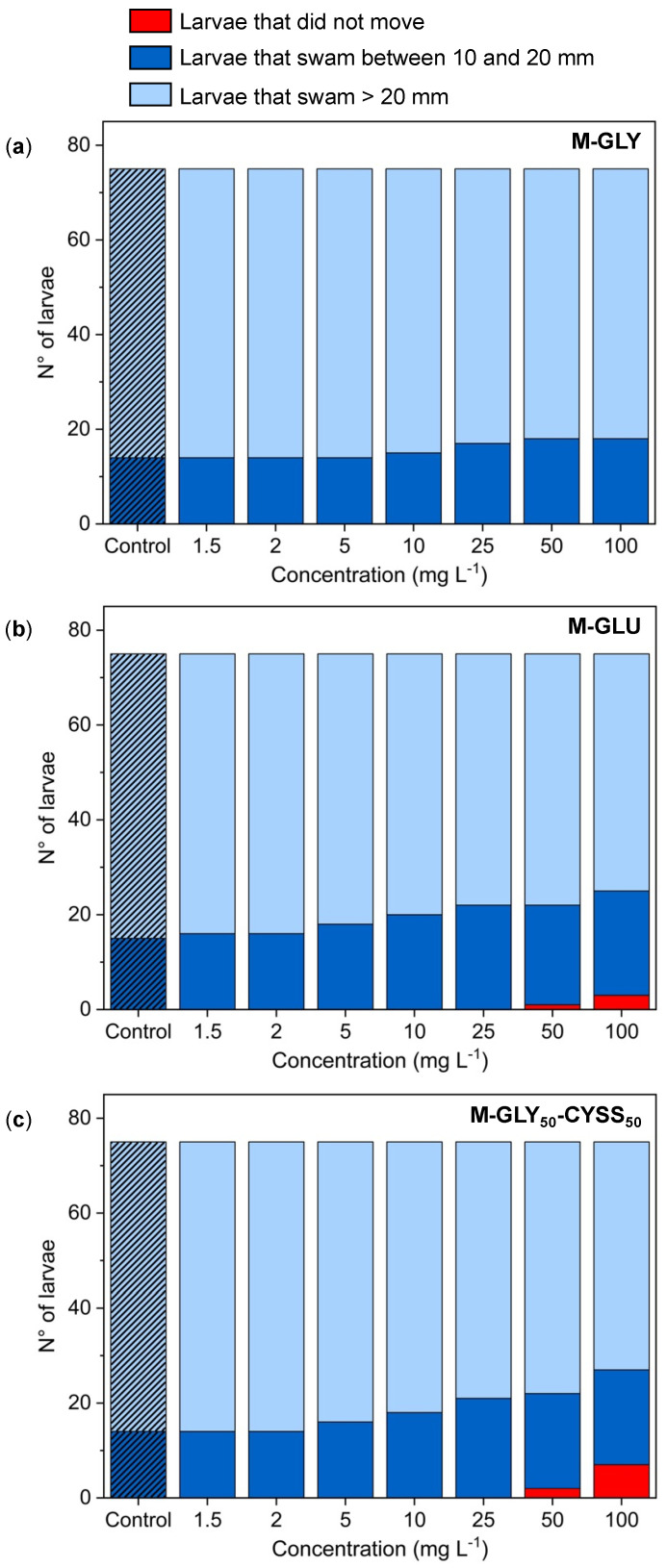
Results of the touch-evoked test performed on zebrafish larvae exposed at 4 hpf to different (**a**) M-GLY, (**b**) M-GLU, and (**c**) M-GLY_50_-CYSS_50_ concentrations. After 72 hpf, the tail of each single larva placed in the center of a Petri dish was gently touched with a smooth pipette tip, and the touch response was observed and categorized as follows: (1) larvae that did not move, i.e., that remained within the circle with 10 mm diameter; (2) larvae that swam between the circles with 10 and 20 mm diameters; and (3) larvae that swam across the outer circle with 20 mm diameter. Results were derived from three independent experiments performed using 25 embryos.

Figure 9 presents the results of this study, comparing the number of larvae that traveled various distances with those that remained immobile for each PAA concentration and the control. Larvae exposed to M-GLY solutions exhibited behavior like the control up to 25 mg L^−1^, with 81% crossing the outer 20 mm diameter circle and none remaining inside the inner 10 mm diameter circle (Table S1). At greater doses, the percentage of larvae that crossed the outer circle slightly reduced to 76%. In M-GLU solutions, a few larvae did not move at the greatest concentration of 100 mg L^−1^. The percentage of larvae that crossed the outer circle reduced to 73 at 10 mg L^−1^, compared to 80 in the control. In M-GLY_50_-CYSS_50_ solutions, the number of larvae that remained immobile at 100 mg L^−1^ increased to around 9%. Meanwhile, the percentage of larvae that crossed the outer circle reduced to 76 at 10 mg L^−1^, compared to 81 in the control. Overall, the results of the touch-evoked response test suggest that none of the PAAs significantly affected the responsiveness of zebrafish larvae up to 10 mg L^−1^ and that the reduction in motility was only mild at greater concentrations in M-GLU and M-GLY_50_-CYSS_50_ solutions.

It is important to note that all PAAs examined in this study are primarily anionic, whereas, among polyelectrolytes, cationic polymers are notably recognized for their toxic properties. It can also be observed that M-GLY, producing the least effect on larvae motility, has only a moderate negative net charge at a pH of 7.0 (Table 1), whereas M-GLU, which exhibited a more pronounced inhibiting effect, has a greater charge density at the same pH (Table 1). Moreover, the M-GLY_50_-CYSS_50_ copolymer, bearing 50% cystine-based units on a molar basis, showed a substantial increase in its inhibitory effect starting from 10 mg L^−1^. This observation is likely due to the specific biochemical activity associated with the disulfide group of cystine. Disulfides are known to participate in a wide range of radical-based synthetic organic and biochemical transformations. Specifically, the reduction in disulfides to their corresponding radical anions, followed by S–S bond cleavage to produce thiyl radicals, play crucial roles in radical-based photoredox transformations [63].

## 4. Conclusions

The ecocompatibility of three α-amino acid-derived polyamidoamines was assessed using the zebrafish (*Danio rerio*) embryo toxicity test, a valuable tool for ecological risk assessments recognized by the European Experimental Substitution Research Center as an alternative animal test. The early stage of embryo development was studied using the fish embryo acute toxicity (FET) test. Embryos showed less than 10% mortality when exposed for 5 days to PAA doses from 1.5 to 500 mg L^−1^. They were therefore classified as non-toxic, non-carcinogenic, and non-dangerous, as it was not possible to identify any LC_50_ value within this concentration range.

Morphological abnormalities were searched for and found in numbers comparable to controls and without PAA dose dependence. In the absence of significant macroscopic defects, microscopic defects affecting the vascular and musculoskeletal systems were searched for using transgenic zebrafish lines expressing a fluorescent protein in selected tissues at two PAA concentrations. Slight alterations were observed, often comparable to those of the control. The neurotoxicity of PAAs was evaluated by analyzing the response of zebrafish larvae subjected to sudden stimuli, both light and tactile, using the light/dark locomotion test and the touch-evoked test. Although slight mobility reduction was observed for intermediate M-GLY concentrations, the touch-evoked response assay allowed clear identification of small behavioral differences among the tested PAAs, ranking M-GLY as the PAA exhibiting the least negative effect on the zebrafish’s neuromotor system. Overall, both tests confirmed that the PAAs under investigation do not show significant neurotoxicity in a wide compositional range.

As a conclusive remark, all the results reported here indicate a low aquatic ecotoxicity of the water-soluble PAAs of interest. This prompts further ecotoxicological studies to rule out possible long-term toxic effects on the environment. Furthermore, this work has paved the way for further insights into the relationship between ecotoxicity and structural characteristics of PAAs. Future studies could focus on evaluating the ecotoxicity of PAAs with a predominant positive charge in the repeating units, considering that cationic polymers are well-recognized as toxic.

## Figures and Tables

**Table 1:** Average charge on PAAs’ repeat units.

PAA	pKa Values	IP ^(a)^	Net Charge at pH 7	Positive/Negative Charge ^(b)^
M-GLY [7]	*pK_a-COOH_* = 1.9 *pK_a-NR3_* = 7.7	4.8	−0.39	0.61
M-GLU [41]	*pK_a-COOH,1_* = 2.32 *pK_a-COOH,2_* = 4.28 *pK_a-NR3_* = 7.78	3.3	−1.14	0.43
M-GLY50-CYSS50 ^(c)^	-	4.9	−0.35	0.72
M-CYSS [42]	*pK_a-NR3,1_* = 2.4 *pK_a-NR3,2_* = 4.0 *pK_a-COOH,1_* = 8.2 *pK_a-COOH,2_* = 12.7	5.0	−0.31	0.84

^(a)^ Isoelectric point. ^(b)^ Data calculated at a pH of 7.5. ^(c)^ Data calculated from the 1:1 contribution of the M-GLY and M-CYSS repeat units.

## Data Availability

The raw/processed data required to reproduce the above findings cannot be shared at this time as the data also form part of an ongoing study.

## Supplementary Materials

The following supporting information can be downloaded at: https://www.mdpi.com/article/10.3390/polym16142087/s1, Figure S1: Scheme of the light/dark locomotion test, Figures S2–S4: ^1^H-NMR spectra of M-GLY, M-GLU, and M-GLY_50_-CYSS_50_, Figures S5–S7: speciation diagrams of M-GLU, M-GLY, and M-CYSS, Figure S8: (a,b) Survival rates of zebrafish embryos at different time points after exposure to several concentrations of M-GLY, M-GLU, and M-GLY_50_-CYSS_50_, Figures S9–S11: Bright-field images of morphological alterations of zebrafish larvae at 72 hpf after M-GLY, M-GLU, and M-GLY_50_-CYSS_50_ exposure; Table S1: Results of the touch-evoked response test.

## Abbreviation

PAA	Polyamidoamine.
REACH	Regulation, evaluation, authorization, and restriction of chemicals.
M	N,N′-Methylenebisacrylamide.
M-GLY	M-glycine-derived polyamidoamine.
M-CYSS	M-cystine-derived polyamidoamine.
M-GLY_50_-CYSS_50_	M-glycine-cystine-derived polyamidoamine.
M-GLU	M-glutamic acid-derived polyamidoamine.
EGFP	Enhanced green fluorescent protein.
*Bmp*	Bone morphogenetic protein.
*Fli1*	Friend leukemia integration 1 transcription factor.
Tg(*fli1*:EGFP)	Transgenic zebrafish line with the endothelial marker *fli1* driving EGFP.
Tg(*Bmp*:EGFP)	Transgenic zebrafish line with the bmp marker driving EGFP.
Hpf	Hours post-fertilization.
OECD	Organization for economic co-operation and development.
LC_50_	50% lethal concentration.
FET	Fish embryo acute toxicity.
ISV	Intersegmental vessel.
DLAV	Dorsal longitudinal anastomotic vessel.
DA	Dorsal aorta.
