# Supplementary material for: Ecotoxicity Assessment of α-Amino Acid-Derived Polyamidoamines Using Zebrafish as a Vertebrate Model
Source: Polymers (Basel). 2024 Jul 22;16(14):2087. doi: 10.3390/polym16142087 (PMC11280761; doi:10.3390/polym16142087)
Supplement: Supplementary file 1 [file polymers-16-02087-s001.zip › polymers-3103078-supplementary.pdf]

Supplementary Materials

Ecotoxicity assessment of α-amino acid-derived polyamidoamines using zebrafish as a vertebrate model

Sofia Treccani,[a] Paolo Ferruti,[a] Jenny Alongi,[a] Eugenio Monti,[b] Daniela Zizioli[b*], Elisabetta Ranucci[a*]

[a] Dipartimento di Chimica, Università degli Studi di Milano, via C. Golgi 19, 20133, Milano, Italy

[b] Dipartimento di Medicina Molecolare e Traslazionale, Università degli Studi di Brescia, viale Europa 11, 25123, Brescia, Italy

* corresponding authors: elisabetta.ranucci@unimi.it, Tel. +390250314132; daniela.zizioli@unibs.it, Tel. +390303717546

Pages S1-S13

Figure S1: Scheme of the light/dark locomotion test.

Figures S2-S4: ^{1}H-NMR spectra of M-GLY, M-GLU and M-GLY$_{50}$-CYSS$_{50}$.

Figures S5-S7: speciation diagrams of M-GLU, M-GLY and M-CYSS.

Figures S8a and **8b:** Survival rates of zebrafish embryos at different time points after exposure to several concentrations of M-GLY, M-GLU and M-GLY$_{50}$-CYSS$_{50}$.

Figures S9-S11: Bright field images of morphological alterations of zebrafish larvae at 72 hpf after M-GLY, M-GLU and M-GLY$_{50}$-CYSS$_{50}$ exposure.

Table S1: Results of the touch-evoked response test.

Locomotor behavior assessment with the light/dark locomotion test

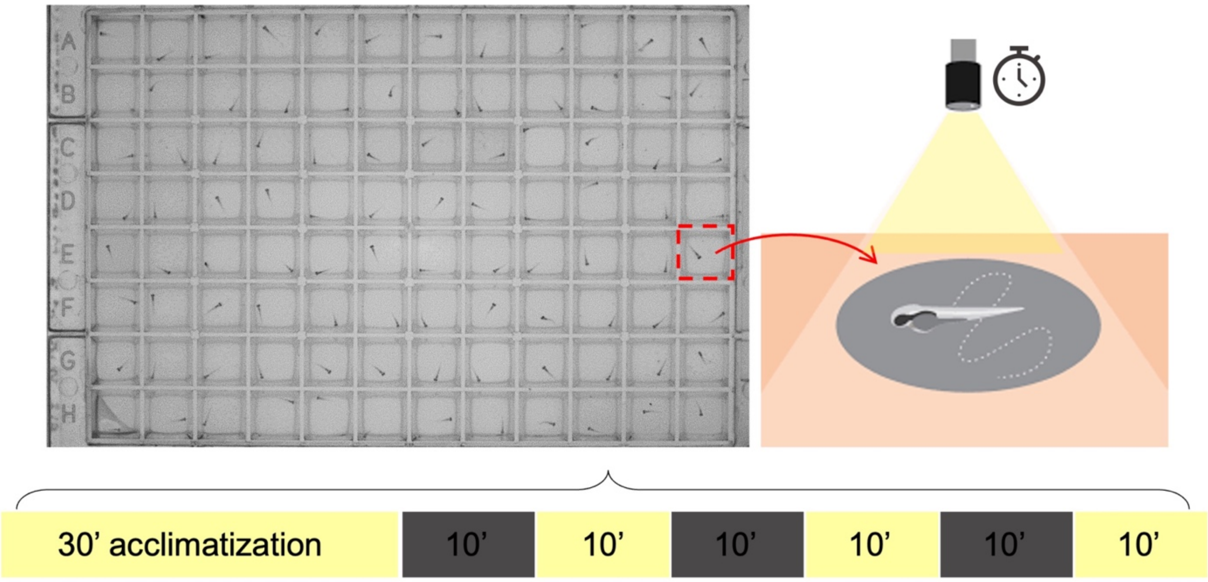

Figure S1. Scheme of the light/dark locomotion test. At 120 hpf, zebrafish larvae were placed in a 96-well plate with one larva per well and placed in a *DanioVision* observation chamber. The test lasted 90 min, of which 30 min light acclimatization followed by 3 cycles of alternating dark/light cycles lasting 10 min per type of exposure.

^{1}H-NMR characterization of PAAs

The chemical structure of M-GLY, M-GLU and M-GLY$_{50}$-CYSS$_{50}$ was assessed by ^{1}H-NMR, collecting spectra in D_2O at pH 4.0 and at 25 °C using a Bruker Advance DPX-400 NMR spectrometer (Milan, Italy) operating at 400.13 MHz. Parameters: scan number 32, relaxation delay, *d1*, 10.0 s, receiver gain automatically measured and set by the instrument.

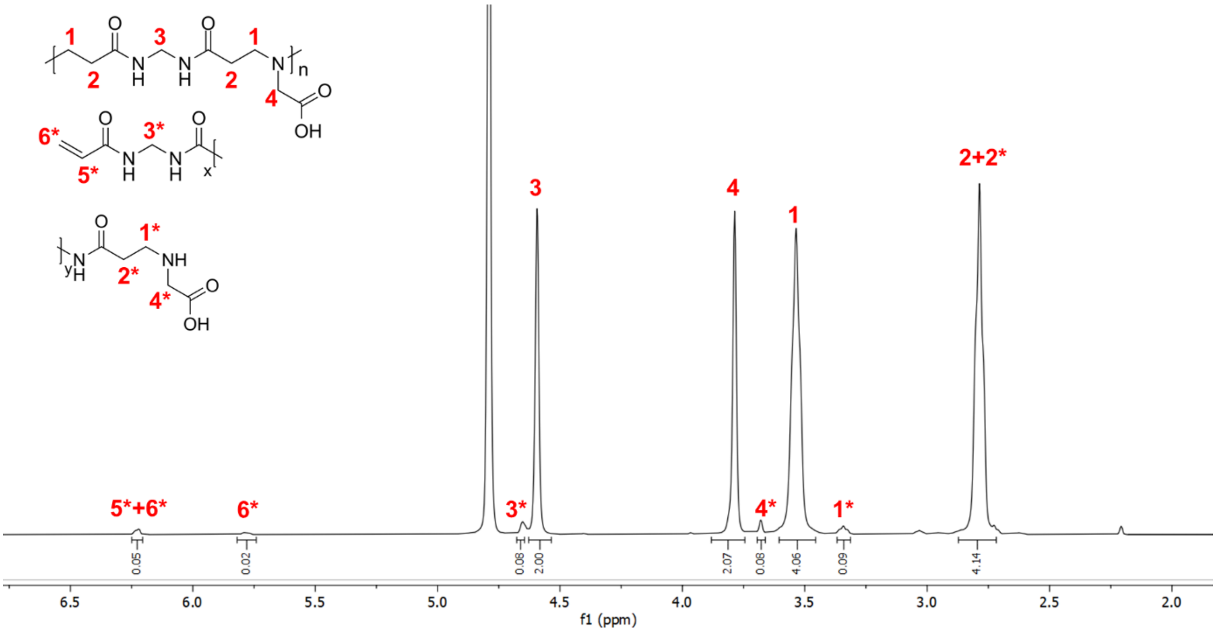

Figure S2. ^{1}H-NMR spectrum of M-GLY.

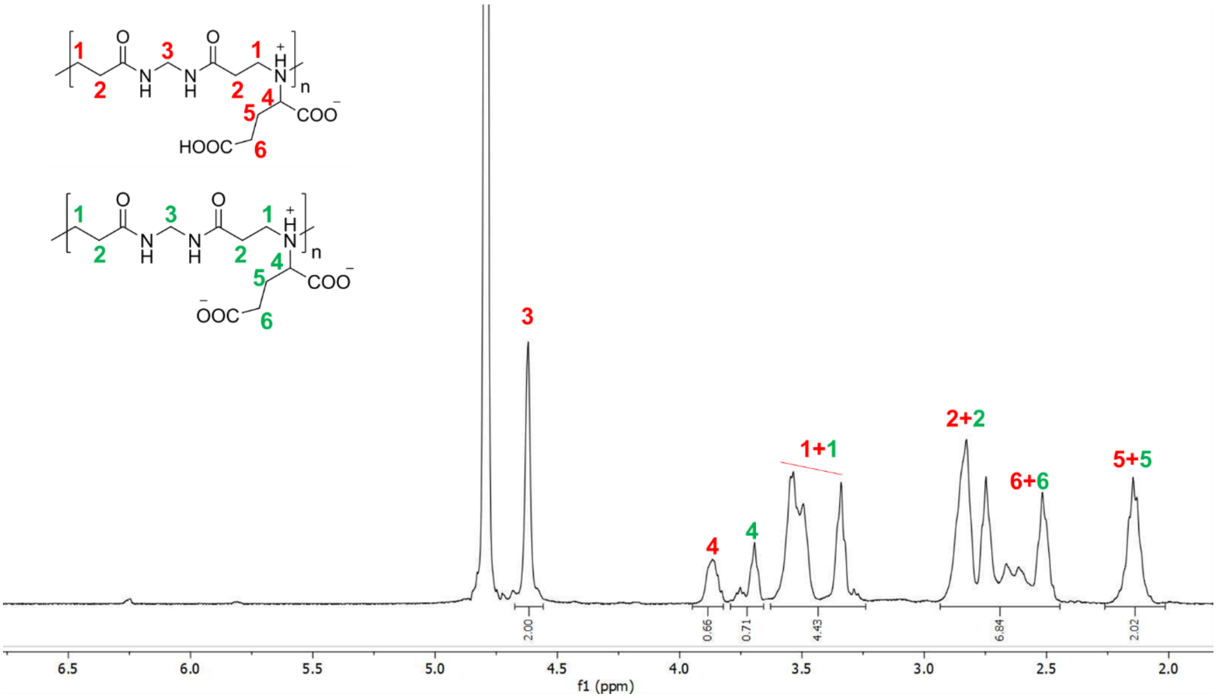

Figure S3. ^{1}H-NMR spectrum of M-GLU.

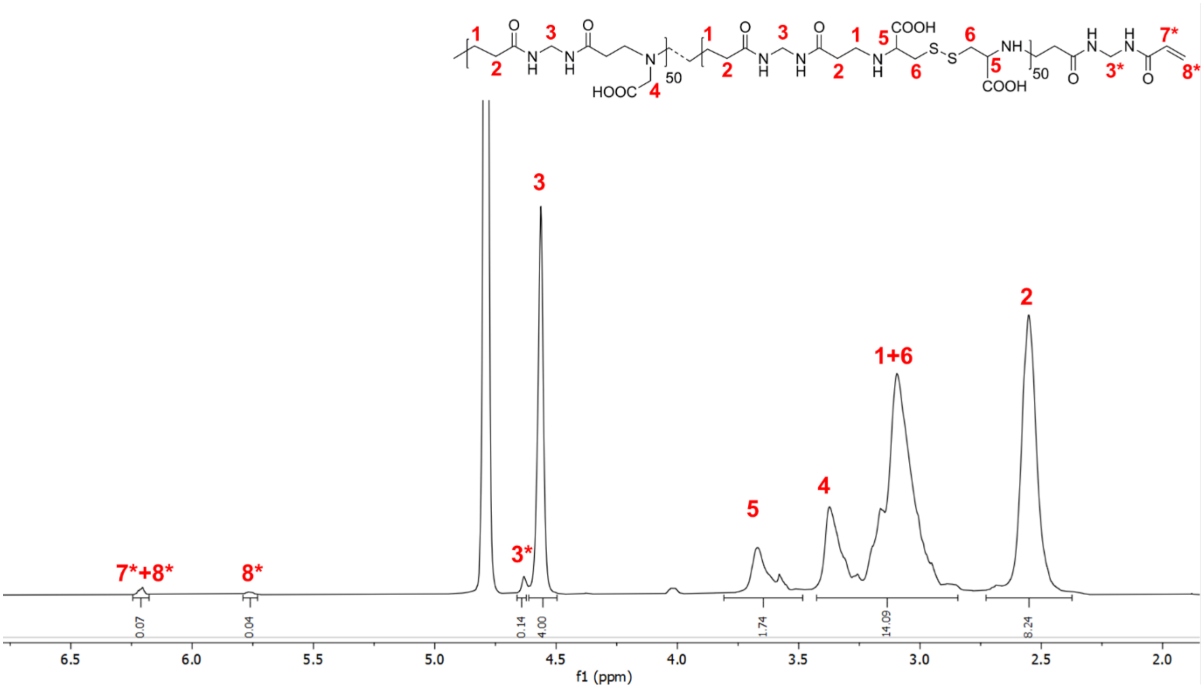

Figure S4. ^{1}H-NMR spectrum of M-GLY$_{50}$-CYSS$_{50}$.

Speciation diagrams of PAAs

M-GLU: pK_a values were calculated in a previous work [1]. Speciation diagrams (Figure S5) were obtained by plotting the concentration fractions (α) of the different ionic species as a function of pH. The concentration fractions of M-GLU were calculated according to the following equations:

- Mass balance: $C_0 = C_{L+} + C_{L0} + C_{L-} + C_{L2-}$
- Equilibrium constants: $K_{a1} = \frac{C_{L0}\, C_{H+}}{C_{L+}}$; $K_{a2} = \frac{C_{L-}\, C_{H+}}{C_{L0}}$; $K_{a3} = \frac{C_{L2-}\, C_{H+}}{C_{L-}}$
- Concentration fractions:

$$C_{L+} = \frac{C_0\, {C_{H+}}^3}{{C_{H+}}^3 + {C_{H+}}^2 K_{a1} + C_{H+} K_{a1} K_{a2} + K_{a1} K_{a2} K_{a3}} = \frac{C_0\, {C_{H+}}^3}{D} \rightarrow \boldsymbol{\alpha}_{\mathbf{L+}} = \frac{C_{L+}}{C_0} = \frac{{C_{H+}}^3}{D}$$

$$C_{L0} = \frac{C_0\, {C_{H+}}^2 K_{a1}}{D} \rightarrow \boldsymbol{\alpha}_{\mathbf{L0}} = \frac{C_{L0}}{C_0} = \frac{{C_{H+}}^2 K_{a1}}{D}$$

$$C_{L-} = \frac{C_0\, C_{H+} K_{a1} K_{a2}}{D} \rightarrow \boldsymbol{\alpha}_{\mathbf{L-}} = \frac{C_{L-}}{C_0} = \frac{C_{H+} K_{a1} K_{a2}}{D}$$

$$C_{L2-} = \frac{C_0\, K_{a1} K_{a2} K_{a3}}{D} \rightarrow \boldsymbol{\alpha}_{\mathbf{L2-}} = \frac{c_{L2-}}{c_0} = \frac{K_{a1} K_{a2} K_{a3}}{D}$$

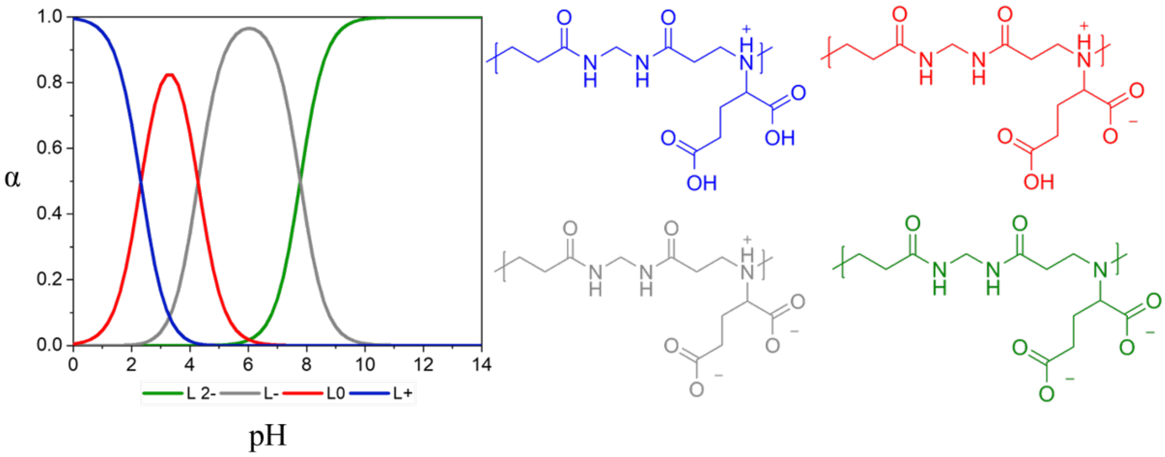

Figure S5. Speciation diagrams and chemical structures of the ionized repeat units of M-GLU.

M-GLY: pK_a values were calculated in a previous work [2]. Speciation diagrams (Figure S6) were obtained by plotting the concentration fractions (α) of the different ionic species as a function of pH. The concentration fractions of M-GLY were calculated according to the following equations:

- Mass balance: $C_0 = C_{L+} + C_{L0} + C_{L-}$
- Equilibrium constants: $K_{a1} = \frac{C_{L0}\, C_{H+}}{C_{L+}}$; $K_{a2} = \frac{C_{L-}\, C_{H+}}{C_{L0}}$
- Concentration fractions:

$$C_{L+} = \frac{C_0\, C_{H+}^{\;2}}{C_{H+}^{\;2} + K_{a1}\, C_{H+} + K_{a1}K_{a2}} = \frac{C_0\, C_{H+}^{\;2}}{D} \rightarrow \boldsymbol{\alpha}_{L+} = \frac{C_{L+}}{C_0} = \frac{C_{H+}^{\;2}}{D}$$

$$C_{L0} = \frac{C_0\, K_{a1}\, C_{H+}}{C_{H+}^{\;2} + K_{a1}\, C_{H+} + K_{a1}K_{a2}} = \frac{C_0\, K_{a1}\, C_{H+}}{D} \rightarrow \boldsymbol{\alpha}_{L0} = \frac{C_{L0}}{C_0} = \frac{K_{a1}\, C_{H+}}{D}$$

$$C_{L-} = \frac{C_0\, K_{a1}K_{a2}}{C_{H+}^{\;2} + K_{a1}\, C_{H+} + K_{a1}K_{a2}} = \frac{C_0\, K_{a1}K_{a2}}{D} \rightarrow \boldsymbol{\alpha}_{L-} = \frac{C_{L-}}{C_0} = \frac{K_{a1}K_{a2}}{D}$$

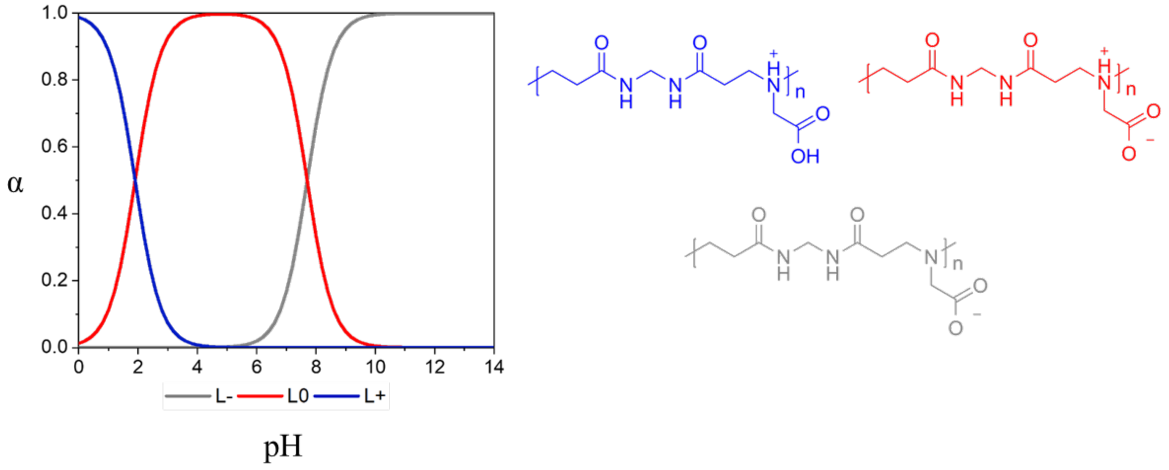

Figure S6. Speciation diagrams and chemical structures of the ionized repeat units of M-GLY.

M-CYSS: K_a values were calculated in a previous work [3]. Speciation diagrams (Figure S7) were obtained by plotting the concentration fractions (α) of the different ionic species as a function of pH. The concentration fractions of M-CYSS were calculated according to the following equations:

- Mass balance: $C_0 = C_{L2+} + C_{L+} + C_{L0} + C_{L-} + C_{L2-}$
- Equilibrium constants: $K_{a1} = \frac{C_{L+}\, C_{H+}}{C_{L2+}}$; $K_{a2} = \frac{C_{L0}\, C_{H+}}{C_{L+}}$; $K_{a3} = \frac{C_{L-}\, C_{H+}}{C_{L0}}$; $K_{a4} = \frac{C_{L2-}\, C_{H+}}{C_{L-}}$
- Concentration fractions:

$$C_{L2+} = \frac{C_0\, C_{H+}^{\;4}}{C_{H+}^{\;4} + C_{H+}^{\;3}K_{a1} + C_{H+}^{\;2}\, K_{a1}K_{a2} + C_{H+}K_{a1}K_{a2}K_{a3} + K_{a1}K_{a2}K_{a3}K_{a4}} = \frac{C_0\, C_{H+}^{\;4}}{D} \rightarrow \boldsymbol{\alpha}_{L2+} = \frac{c_{L2+}}{c_0} = \frac{C_{H+}^{\;4}}{D}$$

$$C_{L+} = \frac{C_0 C_{H+}^{\;3} K_{a1}}{D} \rightarrow \boldsymbol{\alpha}_{L+} = \frac{c_{L+}}{c_0} = \frac{C_{H+}^{\;3} K_{a1}}{D}$$

$$C_{L0} = \frac{C_0\, C_{H+}^{\;2}\, K_{a1}K_{a2}}{D} \rightarrow \boldsymbol{\alpha}_{L0} = \frac{c_{L0}}{c_0} = \frac{C_{H+}^{\;2}\, K_{a1}K_{a2}}{D}$$

$$C_{L-} = \frac{C_0\, C_{H+}K_{a1}K_{a2}K_{a3}}{D} \rightarrow \boldsymbol{\alpha}_{L-} = \frac{c_{L-}}{c_0} = \frac{C_{H+}K_{a1}K_{a2}K_{a3}}{D}$$

$$C_{\text{L2-}} = \frac{C_0\, K_{\text{a1}} K_{\text{a2}} K_{\text{a3}} K_{\text{a4}}}{D} \rightarrow \boldsymbol{\alpha}_{\textbf{L2-}} = \frac{c_{\text{L2-}}}{c_0} = \frac{K_{\text{a1}} K_{\text{a2}} K_{\text{a3}} K_{\text{a4}}}{D}$$

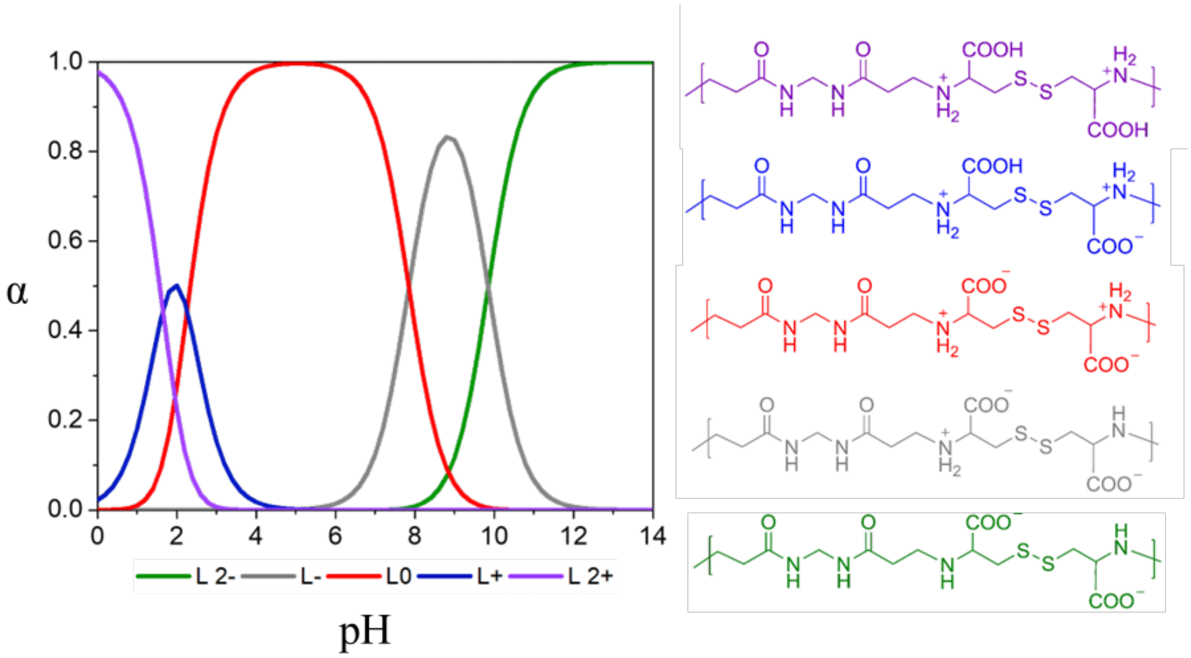

Figure S7. Speciation diagrams and chemical structures of the ionized repeat units of M-CYSS.

Evaluation of survival rate of PAA-exposed zebrafish embryos at different time points

Survival rates of zebrafish embryos were evaluated in the FET test at different time points after exposure to several concentrations of M-GLY, M-GLU and M-GLY_{50}-$CYSS_{50}$ at gastrula stage (4 hpf).

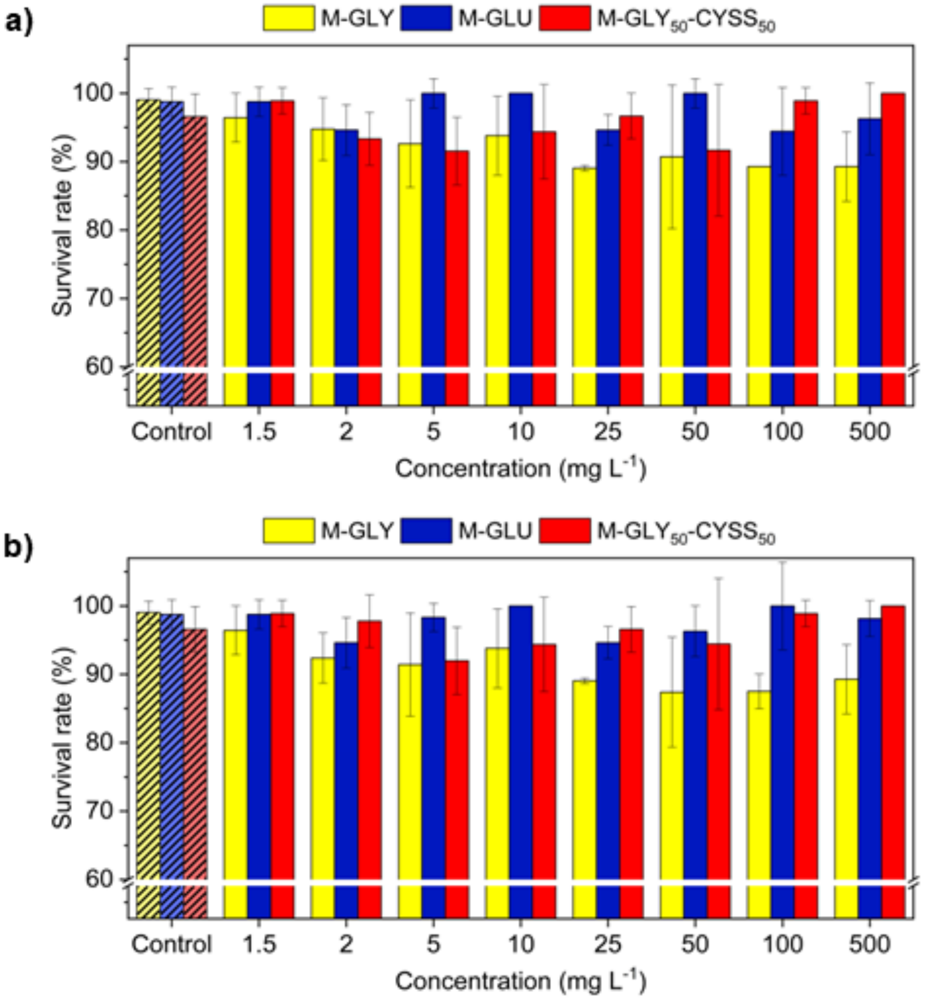

Figure S8. Survival rates of zebrafish embryos at 48 hpf (a) and 72 hpf (b) determined under various concentrations of PAA. Results are reported as the mean ± SD of three independent experiments.

Evaluation of morphological alterations of PAA-exposed zebrafish larvae

Morphological alterations in the FET test were evaluated at 72 hpf through visual inspection of anesthetized zebrafish larvae (0.4% tricaine) from head to tail using Zeiss Axiozoom V13 microscope (Carl Zeiss AG, Oberkochen, Germany). Images were processed with Zen 3.5 (Blue edition). Normal development represents the development of control embryos exposed to fish water.

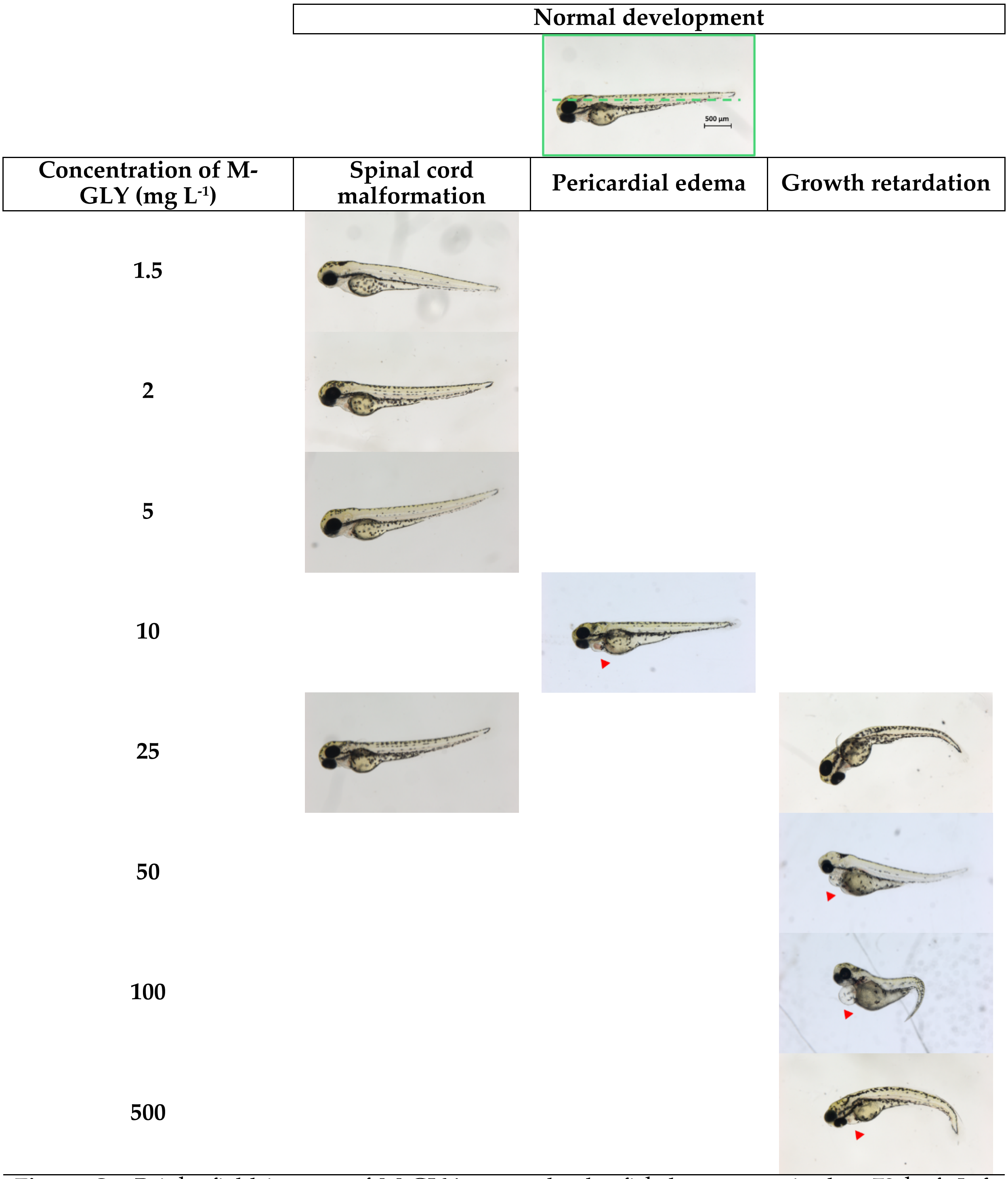

Figure S9. Bright field images of M-GLY exposed zebrafish larvae acquired at 72 hpf. *Left column*: zebrafish larvae with spine deformation. *Middle column*: zebrafish larvae with

pericardial edema (red arrow). *Right column*: zebrafish larvae with growth retardation. Magnification 32X. Scale bar: 500 μm.

	Normal development		
		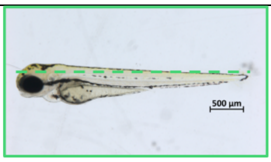	
**Concentration of M-GLU (mg L^{-1})**	**Spinal cord malformation**	**Pericardial edema**	**Growth retardation**
**1.5**		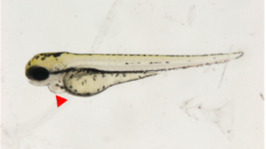	
**2**	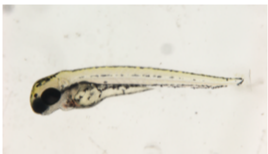		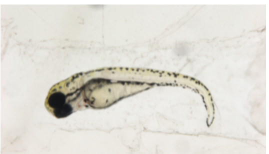
**5**	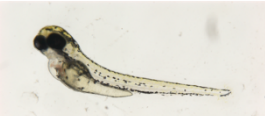		
**10**			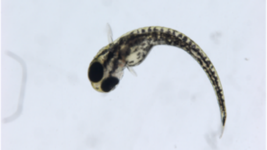
**25**		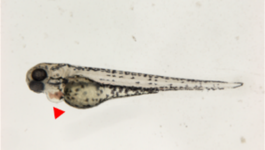	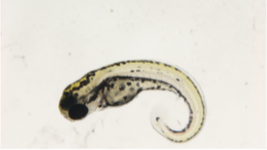
**50**			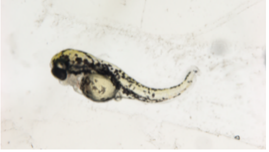
**100**	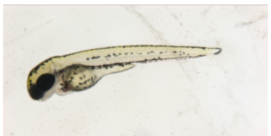	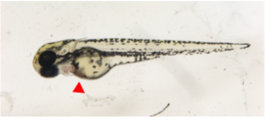	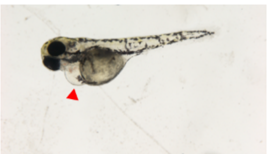
**500**			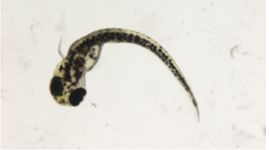

Figure S10. Bright field images of M-GLU exposed zebrafish larvae acquired at 72 hpf. *Left column*: zebrafish larvae with spine deformation. *Middle column*: zebrafish larvae with pericardial edema (red arrow). *Right column*: zebrafish larvae with growth retardation. Magnification 32X. Scale bar: 500 μm.

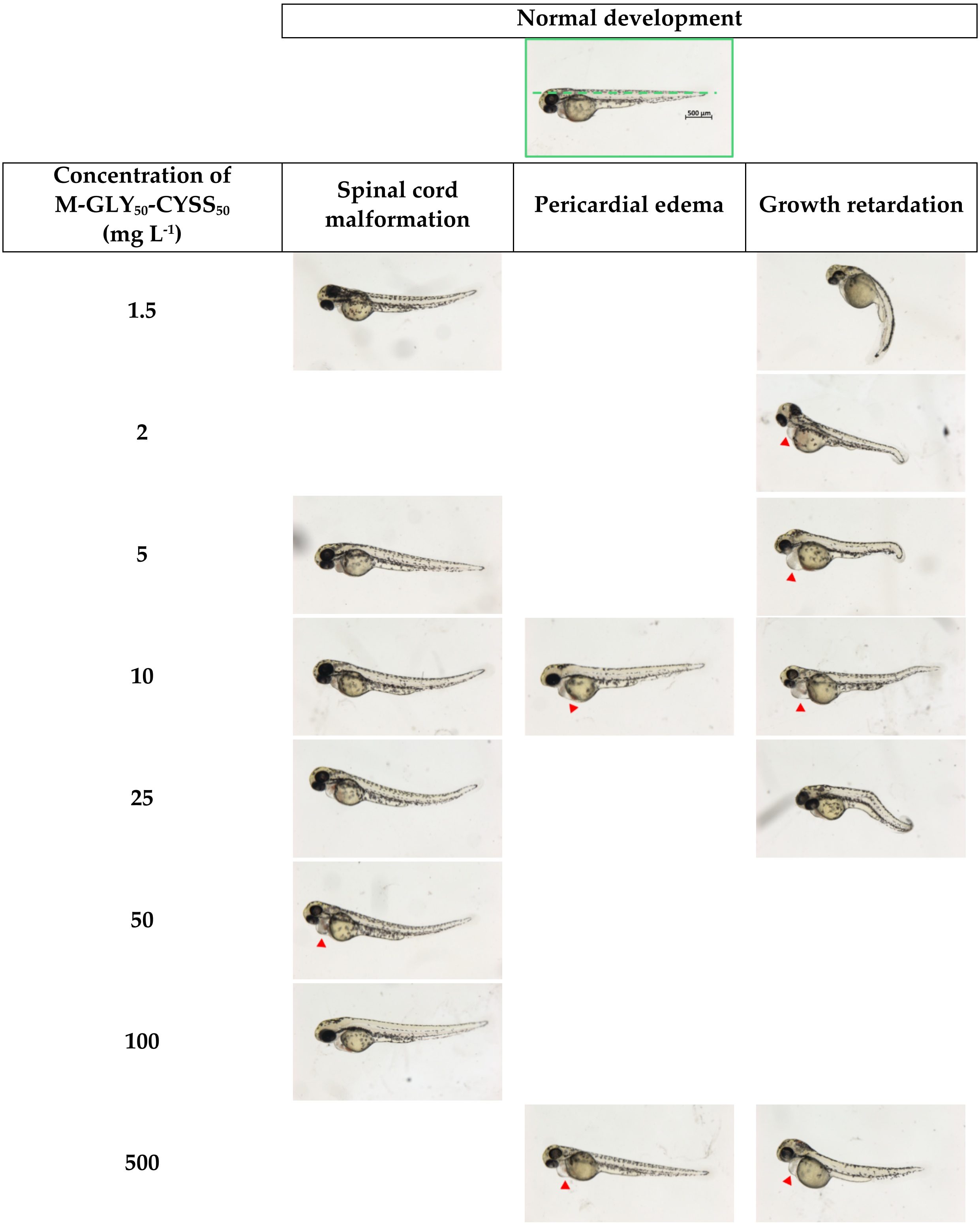

Figure S11. Bright field images of M-GLY$_{50}$-CYSS$_{50}$ exposed zebrafish larvae acquired at 72 hpf. *Left column*: zebrafish larvae with spine deformation. *Middle column*: zebrafish larvae with pericardial edema (red arrow). *Right column*: zebrafish larvae with growth retardation. Magnification 32X. Scale bar: 500 µm.

Touch-evoked response test

AB-strain embryos at 4 hpf were exposed to different PAA concentrations (1.5, 2, 5, 10, 25, 50 100 mg L^{-1}) and fish water as control until 72 hpf. The tail of the embryo was gently touched with a smooth pipette tip and the touch response was observed and categorized as follows: (1) embryos that did not move; (2) embryos that swam < 20 mm; and (3) embryos that swam > 20 mm.

Table S1. Results of the touch-evoked response test.

Concentration (mg L^{-1})	Non-moving embryos (%)	Embryos that swam < 20 mm (%)	Embryos that swam > 20 mm (%)
		**M-GLY**	
Control	-	19 ± 1	81 ± 1
1.5	-	19 ± 1	81 ± 1
2	-	19 ± 1	81 ± 1
5	-	19 ± 1	81 ± 1
10	-	20 ± 4	80 ± 2
25	-	23 ± 2	77 ± 1
50	-	24 ± 1	76 ± 1
100	-	24 ± 1	76 ± 1
		**M-GLU**	
Control	-	20 ± 1	80 ± 1
1.5	-	21 ± 1	79 ± 1
2	-	21 ± 1	79 ± 1
5	-	24 ± 1	76 ± 1
10	-	27 ± 1	73 ± 1
25	-	29 ± 1	71 ± 1
50	1 ± 1	28 ± 0	71 ± 1
100	4 ± 2	29 ± 3	67 ± 1
		**M-GLY_{50}-$CYSS_{50}$**	
Control	-	19 ± 2	81 ± 2
1.5	-	19 ± 2	81 ± 2
2	-	19 ± 2	81 ± 2
5	-	21 ± 3	79 ± 3
10	-	24 ± 3	76 ± 3
25	-	28 ± 2	72 ± 2
50	2 ± 1	27 ± 1	71 ± 1
100	9 ± 3	27 ± 3	64 ± 1

References

1. A. Beduini, F. Carosio, P. Ferruti, E. Ranucci, J. Alongi, Synergism between α-amino acid-derived polyamidoamines and sodium montmorillonite for enhancing the flame retardancy of cotton fabrics, Polym. Degrad. Stab. 225 (2024). https://doi.org/10.1016/j.polymdegradstab.2024.110764.
2. Manfredi, A.; Carosio, F.; Ferruti, P.; Ranucci, E.; Alongi, J. Linear polyamidoamines as novel biocompatible phosphorus-free surface-confined intumescent flame retardants for cotton fabrics. *Polymer Degradation and Stability* **2018**, *151*, 52–64. doi:10.1016/j.polymdegradstab.2018.02.020.
3. Emilitri, E.; Ferruti, P.; Annunziata, R.; Ranucci, E.; Rossi, M.; Falciola, L.; et al. Novel amphoteric cystine-based poly(amidoamine)s responsive to redox stimuli. *Macromolecules* **2007**, *40*(14), 4785–4793. doi:10.1021/ma062115e.
